# In Vitro Propagation and Conservation of *Lavandula stoechas* subsp. *luisieri* and *Pterospartum tridentatum*, Two Important Medicinal and Aromatic Species from Portugal

**DOI:** 10.3390/plants13152124

**Published:** 2024-08-01

**Authors:** Joana Domingues, Anabela Eira, Isa Ramalho, Inês Barrocas, José Carlos Gonçalves

**Affiliations:** 1 Centro de Biotecnologia de Plantas da Beira Interior, Quinta Senhora de Mércules, 6000-191 Castelo Branco, Portugal; joana_mst@hotmail.com (J.D.); anabela.c.eira@gmail.com (A.E.); irramalho2@gmail.com (I.R.); inesbarrocas@gmail.com (I.B.); 2 Polytechnic Institute of Castelo Branco-School of Agriculture (IPCB-ESA), Quinta Senhora de Mércules, 6000-191 Castelo Branco, Portugal; 3 Research Center for Natural Resources, Environment and Society (CERNAS), Polytechnic Institute of Castelo Branco, Av. Pedro Álvares Cabral, 12, 6000-084 Castelo Branco, Portugal

**Keywords:** ex vitro rooting, micropropagation, slow-growth conservation, sucrose

## Abstract

*Lavandula stoechas* subsp. *luisieri* and *Pterospartum tridentatum* are two valuable aromatic and medicinal plants. Their biometric and morphological parameters, such as the number of new shoots, length of the longest shoot, multiplication rate, and fresh weight, were evaluated using the multiplication MS medium protocol. The rooting protocols involved immersing the explants in IBA (1 g L^−1^) and a commercial IBA (3.3 g L^−1^) preparation (Clonex^®^). Slow-growth conservation assays were carried out using two different sucrose concentrations (15 g L^−1^ and 30 g L^−1^), and a control, with the cultures kept at 4 °C for 12 months. The multiplication rate for *L. stoechas* subsp. *luisieri* was 6.8, and that of *P. tridentatum* was 13.3, achieved using the MS medium supplemented with 0.2 mg L^−1^ BAP, 1 mg L^−1^ BAP, and 0.5 mg L^−1^ IBA. The application of Clonex^®^ showed the best ex vitro rooting results in *L. stoechas* subsp. *luisieri* (77%) and *P. tridentatum* (90%). In the slow-growth conservation assays, at 4 °C, in darkness for 12 months, an excellent survival rate was achieved in *L. stoechas* subsp. *luisieri* (>80%) and *P. tridentatum* (>90%), even at the reduced sucrose concentration. This study demonstrates the effectiveness of in vitro multiplication and ex vitro rooting protocols for two valuable aromatic and medicinal plants. These findings are significant for the ex situ conservation of these species, as they provide effective long-term preservation and utilization strategies.

## 1. Introduction

Ecological and phenological changes in flora and fauna due to climate change are currently visible. Global warming significantly impacts the phenology and survival of many species, resulting in changes in plants’ phenology and increased risks to survival. These changes can potentially disrupt ecosystems, alter evolutionary trajectories, and present significant conservation challenges. Continued research and proactive conservation strategies are critical for mitigating these effects and preserving biodiversity in a warming world. It is estimated that about 22,000 plants worldwide are included on the Red List of the International Union for Conservation of Nature and Natural Resources (IUCN), as Near-Threatened, Vulnerable, Endangered, Critically Endangered, Extinct in the Wild, or Extinct [1]. The IUCN classifies *Pterospartum tridentatum* as Least Concern, but there is no information on the status of *Lavandula stoechas* subsp. *luisieri*. Climate change and the industrialization and urbanization of societies have negatively impacted the endemic vegetation in each region worldwide. Due to these critical situations, developing plant conservation strategies is crucial. Applying in vitro conservation methodologies, such as micropropagation has yielded excellent species preservation results. Micropropagation can ensure large-scale production under controlled conditions in a short period, without negative impacts on habitats [2]. In vitro culturing is a versatile and powerful tool in agriculture and biotechnology. It can efficiently produce large numbers of uniform and disease-free plants, making it an invaluable technique for improving crop productivity and sustainability.

Furthermore, the controlled environment of in vitro culturing aids in the production of valuable secondary metabolites and the advancement of genetic engineering and functional genomics research, all of which contribute significantly to the development of innovative agricultural and biotechnological solutions [3]. Among the micropropagation techniques usually used, the proliferation of axillary meristems is the most suitable for plant cloning [4]. Short-term conservation consists of the maintenance of in vitro cultures in active growth, which involves the transference of the cultures to a new medium for a short period. However, this methodology is a laborious and costly approach.

On the other hand, if the objective is to preserve the culture, slow-growth storage is a great strategy for the long-term conservation of in vitro cultures. Slow-growth conservation is a simple in vitro method that permits species conservation from 6 months to 5 years, depending on the species [5]. The slow-growth conditions can be obtained by chemical and physical approaches, such as medium alterations, e.g., reduced sucrose concentration, osmotic agents, reduced oxygen, reduced temperature, and/or light [6]. All of these factors influence the in vitro growth of the cultures to different degrees and can also have synergetic effects [7]. Applying these stress conditions reduces subcultures and consequently reduces labor time and costs. Despite the advantages of this technique, it has some disadvantages, such as the need for storage space, as well as somaclonal risks. [8].

*Lavandula stoechas* subsp. *luisieri* (Lamiaceae family) and *Pterospartum tridentatum* (Fabaceae family) are two important endemic species of the Portuguese flora that are frequently mentioned in ethnobotanical studies [9]. Their extracts and essential oils have shown significant results in therapeutic, pharmaceutical, cosmetic, and culinary applications [9,10,11,12]. The geographical distribution of *L. stoechas* subsp. *luisieri* is mainly in the central and southern parts of Portugal and Spain. Regarding the *L. stoechas* subsp. *luisieri* essential oil, the main volatile compounds are irregular monoterpenoids with cyclopentenic structures, namely necrodane derivatives such as *trans*-α-necrodol and *trans*-α-necrodyl acetate [12,13,14]. Due to this volatile composition, its biological activities have been excellent compared to other species [14,15]. The extracts of *P. tridentatum* have also shown promising results in anti-inflammatory responses due to its flavonolic profile [11,16].

Micropropagation studies on these species are scarce, and their in vitro conservation is reported for the first time in this manuscript. The in vitro multiplication of *L. stoechas* was described by Nobre [17], who mentioned that the multiplication phase was carried out using single-node explants cultured on a basal medium containing Margara N30K macrosalts [18], along with microsalts and vitamins of Murashige and Skoog (MS) [19], supplemented with 40 mg L^−1^ of adenine hemisulfate and 0.01 mg L^−1^ of 1-naphthaleneacetic acid (NAA) [17]. In the same study, the author also showed the best rooting conditions on basal medium containing 1 mg L^−1^ NAA [17]. A few years later, some authors reported the best multiplication rate in *L. viridis* with 0.15 mg L^−1^ of 6-benzylaminopurine (BAP) in MS medium with macronutrients at half strength [20,21]. In 2010, Zuzarte et al. reported a great multiplication rate in *L. pedunculata* with 0.25 mg L^−1^ of BAP on MS medium. The authors also reported the rooting of this species on an MS medium supplemented with 10 mg L^−1^ of ascorbic acid without growth regulators [22]. The information on *P. tridentatum* micropropagation is scarce—only a PhD thesis from Portugal, which reported the best multiplication rate in this species on MS medium supplemented with 1 mg L^−1^ of BAP and 0.5 mg L^−1^ of indole-3-butyric acid (IBA) [23]. The MS basal medium has also been reported in other Fabaceae family species [24,25].

Therefore, given the potential of these species for healthcare, pharmaceutical, and food industries, along with the threat to their survival in their natural habitats due to climate change, it is crucial to find ways to preserve them. Thus, the main goals of the present study are to describe a micropropagation protocol for *L. stoechas* subsp. *luisieri* and *P. tridentatum* and to investigate the behavior of these species in a slow-growth conservation process.

## 2. Results

### 2.1. In Vitro Establishment and Multiplication

**Figure 1:**
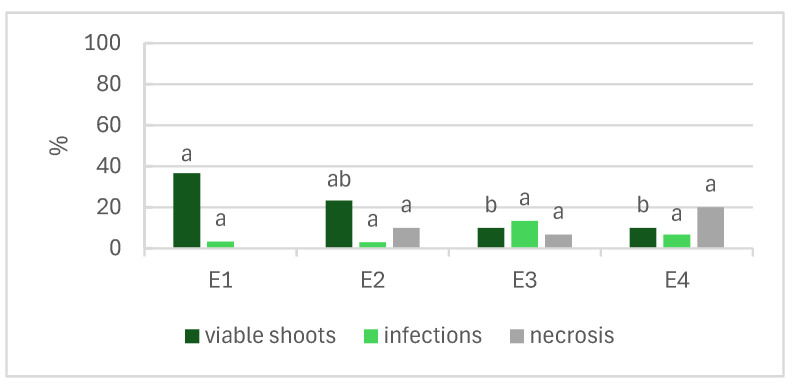
Effects of the concentration and time of exposure of NaOCl in disinfection of *Lavandula stoechas* subsp. *luisieri* explants. E1: 1% NaOCl for 10 min; E2: 1% NaOCl for 15 min; E3: 2% NaOCl for 10 min; E4: 2% NaOCl for 15 min. Different letters indicate significant differences among the parameters (*p* ≤ 0.05).

Figure 1 shows the effects of the NaOCl concentration and time of exposure in establishing *L. stoechas* subsp. *luisieri* explants. According to the results, the increment of the NaOCl concentration had a negative impact on the explants. In the E3 and E4 treatments, we observed that less than 10% of the shoots were viable, and necrosis increased in treatment E4 with increasing exposure to NaOCl. The E1 treatment (1% NaOCl for 10 min) produced the highest number of viable shoots (37%), followed by E2 (1% NaOCl for 15 min), with 23% viable shoots. 

Table 1 shows the results of the biometric parameters in the multiplication phase of 50 days for *L. stoechas* subsp. *luisieri* and *P. tridentatum* explants. In *L. stoechas* subsp. *luisieri*, the average number of new shoots was 3.3, the longest shoot was 4.9 cm long, the multiplication rate was 6.8, and the fresh weight was 260 mg. No hyperhydricity or mortality was observed. In the multiplication phase of *P. tridentatum*, the biometric parameters showed great values: the average number of new shoots was 6.8, the length of the longest shoot was 5.1 cm, and the multiplication rate was 13.

### 2.2. Ex Vitro Rooting

Three treatments were performed in the rooting experiments of the two species. Table 2 shows their effects. In *L. stoechas* subsp. *luisieri*, no significant differences were found in the rooting rate, with values of 67% for the control and 77% for the Clonex treatment. However, significant differences were found for the number and length of the longest root, with the best results achieved with Clonex, with 15 and 8.1 cm, respectively. In *P. tridentatum*, the rooting rate value was similar in the IBA (83%) and Clonex^®^ (90%) treatments, but significantly different from the control. In the Clonex^®^ rooting treatment, the average number of roots was 2.1, which was similar to the IBA rooting treatment and statistically different from the control (0.9). The longest root length did not differ between the IBA and control treatments.

### 2.3. Slow-Growth Conservation 

**Figure 2:**
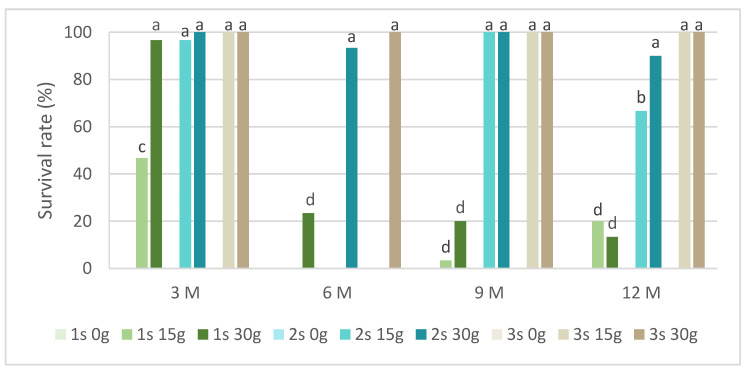
The survival rate of *Lavandula stoechas* subsp. *luisieri* explants over experiment time. 1 s: first subculture; 2 s: second subculture; 3 s: third subculture; 3 M: 3 months; 6 M: 6 months; 9 M: 9 months; 12 M: 12 months. Different letters correspond to statistical differences (*p* < 0.05).

In slow-growth conservation experiments, the time of slow-growth exposure and two sucrose concentrations were tested. Figure 2 presents the survival rate in *L. stoechas* subsp. *luisieri* explants for three 30-day successive subcultures after being removed from slow-growth conditions and starting to grow in standard physical conditions. We observed that in all evaluation times, the explants cultured in a medium without sucrose did not survive. In the shortest evaluation time (after 3 months), the first subculture showed the highest survival rate values, above 40%. Over time, the first subculture was mostly below 20%; however, the *L. stoechas* subsp. *luisieri* explants recovered in the second and third subcultures. The survival results after 6 months with 15 g of sucrose are surprising compared to those obtained after 9 and 12 months, even though they were also low (3.3 and 20%, respectively). We can admit an experimental error if a physiological explanation is not plausible. 

However, both sucrose concentrations showed a positive explant response at the ninth and twelfth months. The first subcultures were affected, with a survival rate close to 20%. The third subcultures at the third, ninth, and twelfth months did not show differences between 15 g L^−1^ and 30 g L^−1^ sucrose.

**Figure 3:**
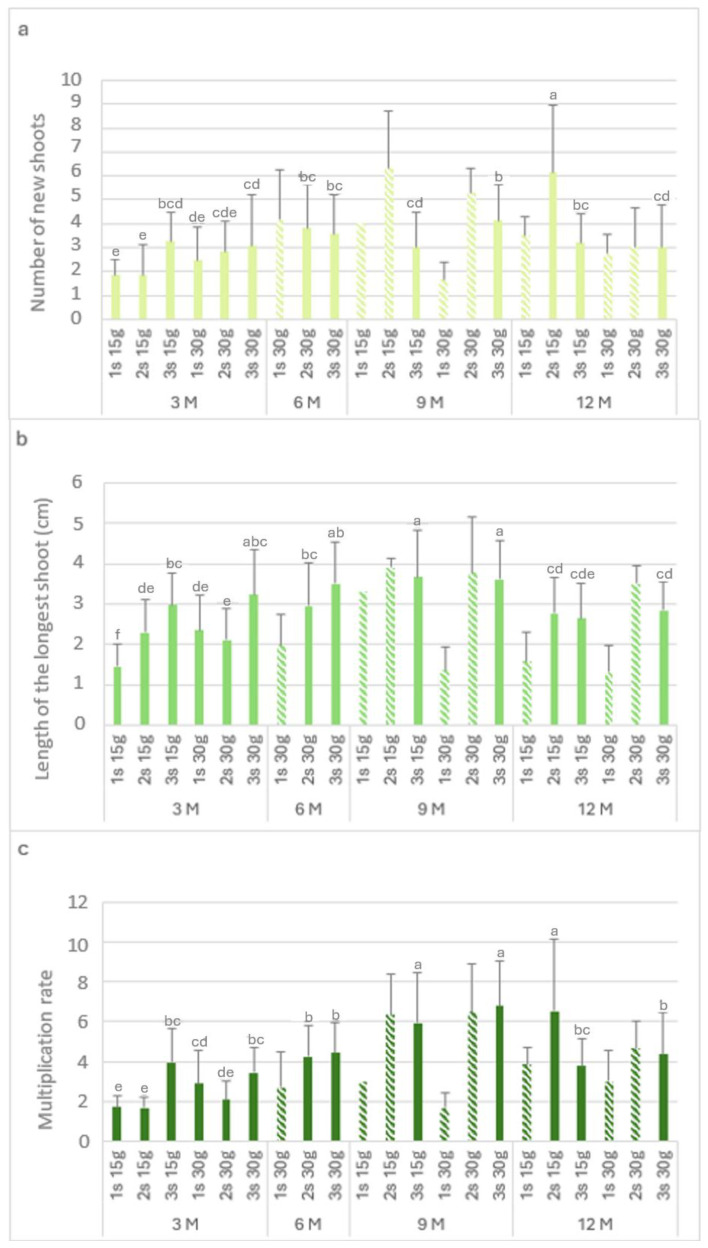
The number of new shoots (**a**), the length of the longest shoot (**b**), and the multiplication rate (**c**) of *Lavandula stoechas* subsp. *luisieri* explants from first (1 s), second (2 s), and third (3 s) subcultures in standard conditions after cold storage for 3, 6, 9 and 12 months. 3 M: 3 months; 6 M: 6 months; 9 M: 9 months; 12 M: 12 months. Bars with a pattern of oblique lines were not considered for statistical analysis. Different letters correspond to statistical differences according to Duncan’s test (*p* < 0.05).

Figure 3 shows the biometric parameters of the *L. stoechas* subsp. *luisieri* explants in the slow-growth conservation during the experiment time. 

Figure 3a presents the number of new shoots (Nns) over time. The highest Nns value (6.2) was observed after 12 months with 15 g L^−1^ sucrose in the second subculture, followed by the third subculture with 30 g L^−1^ sucrose after 9 months. The explants showed a recovery in the Nns values in the second and third subcultures. After 9 months at 4 °C, either with the 15 g L^−1^ sucrose or 30 g L^−1^ sucrose concentration, there was an increase in the Nns values in the second subculture (>5) compared to the first subculture (<4). Finally, after 12 months, the highest Nns value (6.2) was obtained with 15 g L^−1^ sucrose; this applied to all the subcultures. 

The length of the longest shoot (Ls) is presented in Figure 3b. In explants kept in cold storage for 3 months, the highest value was verified with 30 g L^−1^ sucrose in the third subculture (3.2 cm), which was not statistically different from the third subculture with 15 g L^−1^ sucrose (3 cm). After 6 months with 30 g L^−1^ sucrose, an increase was observed in the subcultures. In the ninth month, the highest values were verified in the third subculture with 15 g L^−1^ and 30 g L^−1^ sucrose, with 3.7 cm and 3.6 cm, respectively.

In explants kept in cold storage for 12 months, the first subculture length values varied from 1.3 to 1.6 cm, respectively, with 15 g L^−1^ and 30 g L^−1^ sucrose; however, the values increased in the second and third subcultures to 2.8 cm. With 15 g L^−1^ sucrose, the multiplication rate values (Figure 3c) in the first and second subcultures were low in the explants that had been stored (in cold conditions) for 3 months. At the ninth month, the highest length with 30 g L^−1^ sucrose was observed in the third subculture (6.8). After 12 months in cold conservation, the explants showed multiplication rate values above 3. With 30 g L^−1^ sucrose, the multiplication rate values were round 4, higher than the main values obtained at the third month.

**Figure 4:**
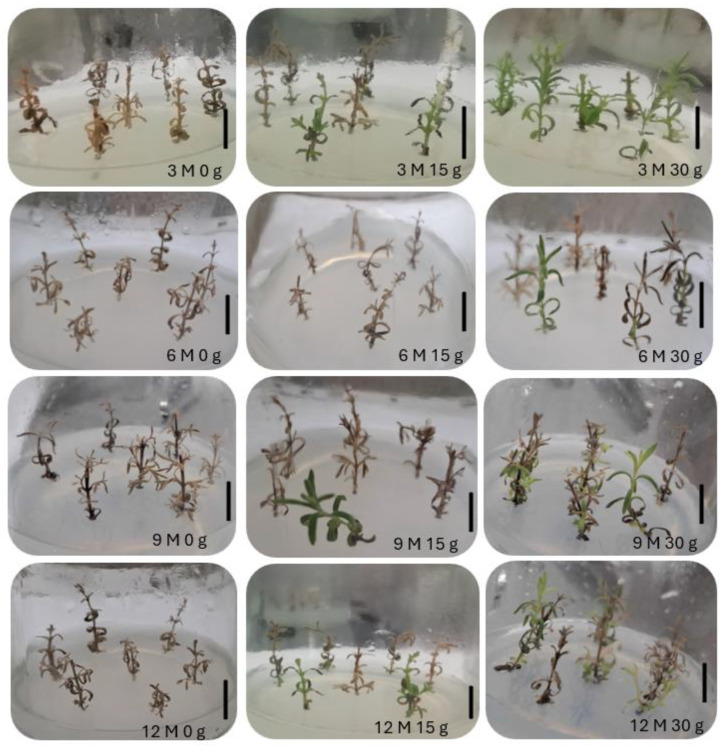
Morphological appearance of *Lavandula stoechas* subsp. *luisieri* explants in standard conditions after cold storage for 3, 6, 9, and 12 months (3 M: 3 months; 6 M: 6 months; 9 M: 9 months; 12 M: 12 months) and sucrose concentration (0 g L^−1^, 15 g L^−1^, and 30 g L^−1^). The bar corresponds to 1 cm.

Figure 4 shows the morphological appearance of *L. stoechas* subsp. *luisieri* explants after being kept at a cold temperature (4 °C) at each time of analysis during the slow-growth assay.

**Figure 5:**
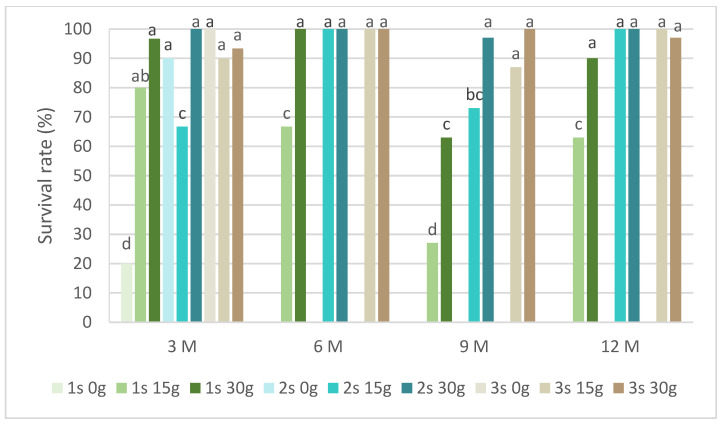
The survival rates of *Pterospartum tridentatum* explants over the experiment time. 1 s: first subculture; 2 s: second subculture; 3 s: third subculture; 3 M: 3 months; 6 M: 6 months; 9 M: 9 months; 12 M: 12 months. Different letters correspond to statistical differences (*p* < 0.05).

Figure 5 presents the survival rates of *P. tridentatum* during the slow-growth assay. Only explants preserved in cold storage for 3 months survived in the medium without sucrose. Even without sucrose, the second and third subcultures recovered survival rates above 80%. In the same conservation time, all subcultures and sucrose concentrations showed a survival rate above 60%, with the 30 g L^−1^ sucrose in the first subculture achieving 97%. In the first subcultures, the survival rate of the 15 g L^−1^ sucrose treatment was always inferior compared to the 30 g L^−1^ sucrose treatment. At 9 months, this value was 27% compared to 63%, respectively, for the 15 g L^−1^ and 30 g L^−1^ sucrose treatments. Generally, all subcultures at both sucrose concentrations showed a survival rate above 80%, even after 12 months of cold conservation.

**Figure 6:**
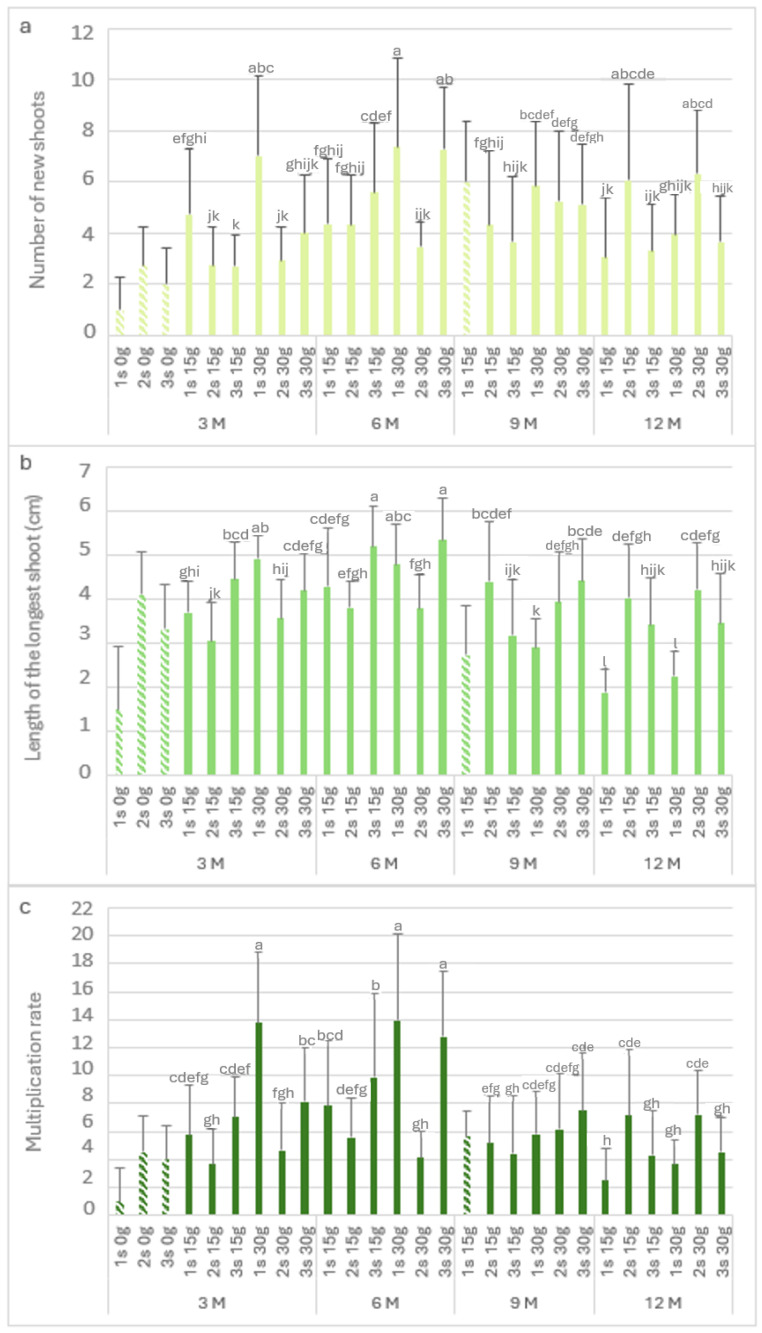
The number of new shoots (**a**), the length of the longest shoot (**b**), and the multiplication rate (**c**) of *Pterospartum tridentatum* explants from the first (1 s), second (2 s), and third (3 s) subcultures in standard conditions after cold storage for 3, 6, 9 and 12 months. 3 M: 3 months; 6 M: 6 months; 9 M: 9 months; 12 M: 12 months. Bars with a pattern of oblique lines were not considered for statistical analysis. Different letters correspond to statistical differences according to Duncan’s test (*p* < 0.05).

Figure 6 shows the biometric parameters of the *P. tridentatum* explants in the slow-growth conservation period. The highest value of Nns (7.4) was observed after 6 months with 30 g L^−1^ sucrose in the first subculture (Figure 6a), followed by the third subculture (7.3) after 6 months of cold storage. It was also not statistically different from the first subculture with 30 g L^−1^ sucrose at 3 months of cold storage (7). At 3 months of cold storage, the Nns of the explants without sucrose ranged from 1 to 2.8. At 15 g L^−1^ and 30 g L^−1^ sucrose, the first subculture produced between 4.8 and 7 new shoots per explant, observing a decrease in the second and third subcultures. Even after 12 months in cold storage, the explants showed high values of Nns; it was observed to be a recovery in the second and third subcultures, with values close to six new shoots in the second subculture at both sucrose concentrations. 

Regarding the length of the longest shoot (Figure 6b), in the absence of sucrose, the first subculture showed values of around 1.5, recovering in the second and third subcultures (>3 cm). The longest shoots were observed after 6 months in the third subcultures at both sucrose concentrations, with 5.2 and 5.3 cm for 15 g L^−1^ and 30 g L^−1^ sucrose, respectively. After 12 months in cold storage, the first subculture at 15 g L^−1^ and 30 g L^−1^ sucrose showed short lengths of 1.9 and 2.2, respectively. However, the longest shoots values (greater than 3 cm) were observed in the consecutive subcultures. Figure 6c shows the multiplication rate, and it is possible to observe the influence of the lack of sucrose; without this carbon resource, the first subculture showed the lowest multiplication rate (1). However, there was a significant increase in the second and third subcultures, with a multiplication rate of 4. The highest value was observed in third month with 30 g L^−1^ sucrose (first subculture), with a value of 13.8. Even after 12 months in cold storage, the multiplication rate in the second subculture for both sucrose concentrations was close to 7.

**Figure 7:**
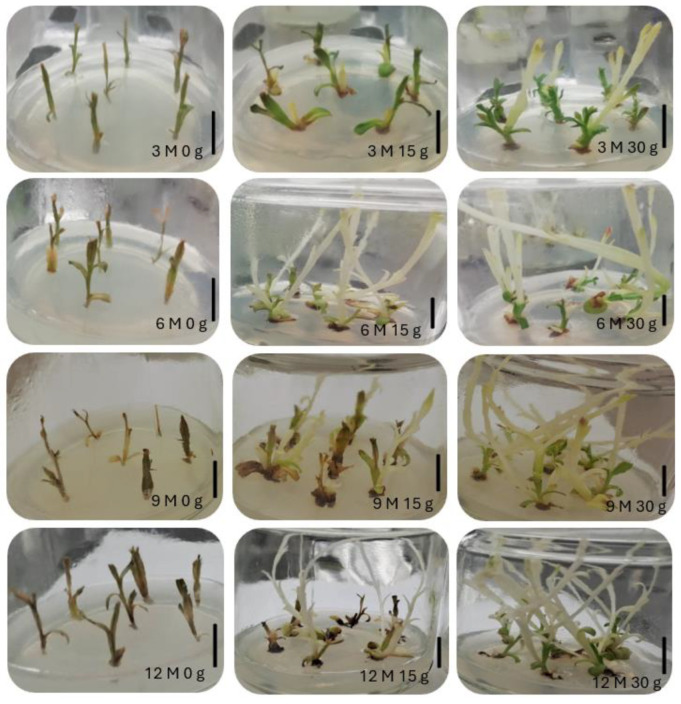
Morphological appearance of *Pterospartum tridentatum* explants in standard conditions after cold storage for 3, 6, 9 and 12 months (3 M: 3 months; 6 M: 6 months; 9 M: 9 months; 12 M: 12 months) and sucrose concentration (0 g, 15 g, and 30 g). The bar corresponds to 1 cm.

Figure 7 presents the morphological appearance of *P. tridentatum* explants after being kept at a cold temperature (4 °C) for 3 months during the slow-growth assay. In this species, all explants, regardless of analysis time and with 15 g L^−1^ and 30 g L^−1^ sucrose, showed etiolated explants because of light absence. However, after the first subculture in light conditions, the explants restored their green chlorophyll color. 

## 3. Discussion

The in vitro establishment of *L. stoechas* subsp. *luisieri* included four disinfection treatments with different NaOCl concentrations and durations of exposure. According to the results, it is possible to state that the increase in the NaOCl concentration had a negative effect on the shoots. The lowest percentage of viable shoots was observed in treatments E3 and E4 with 2% NaOCl, with values below 10%, increasing the necrosis rates by 7 and 20%, respectively. The time of exposure and concentration of the disinfecting agent are two critical factors in this step. However, the contamination level of the mother plant is also crucial for disinfection success. In diverse cultures, the contamination rate could be high, namely in *L. viridis*, which was reported to have a contamination rate of 50% [20]. In this step, it is also crucial to ensure that all explants are exposed equally to the disinfected agent, ensuring some agitation to promote exposure to the entire explant. In *L. pedunculata* in vitro establishment, Zuzarte et al. reported a viable shoot percentage above 80% [22]. The effectiveness of the in vitro establishment, in addition to the factors related to the disinfection process, may also be influenced by the biotic factors of the plant, namely the phenological phase and the type of segments used [2]. According to our results, the contamination levels could be attributed to the plant’s natural contamination and the time of exposure or concentration of the disinfectant agent.

The shoot multiplication step was performed in both species, and their biometric parameters were registered. The number of new shoots, the length of the longest shoot, and the multiplication rate are essential parameters for verifying the performance and health of the culture. 

In *L. pedunculata* multiplication, Zuzarte et al. reported low shoot length values ranging from 1.9 to 2.6 cm, with 1 to 4.1 shoots per explant. Moreover, the authors noticed that intermediate BAP concentrations (0.25 mg L^−1^) were more effective than low or high concentrations (0.1 and 0.5 mg L^−1^, respectively) [22]. The best multiplication rate and the longest shoot length in *L. viridis* were obtained in MS medium with the macronutrient at half strength and 0.15 mg L^−1^ BAP, with 11.7 and 4.4 cm, respectively [20]. The number of new shoots in *P. tridentatum* in vitro multiplication was 6.8, the longest shoot length was 5.1 cm, the multiplication rate was 13.3, and the fresh weight was 340 mg. The information on *P. tridentatum* in vitro multiplication is scarce; only Coelho [23] studied different culture mediums and growth regulators. The author showed that the best conditions were MS supplemented with 1 mg L^−1^ BAP and 0.5 mg L^−1^ IBA, with 3.4 new shoots, a longest shoot length of 3.8 cm, and a 7.7 multiplication rate [23]. The application of MS medium was also reported in the Fabaceae family, supplemented with cytokinins and auxins [24,25].

The rooting process could be performed in in vitro or ex vitro conditions; in this work, rooting was performed in ex vitro conditions for both species. The percentage of root induction for *L. stoechas* subsp. *luisieri* ranged from 73% to 77% for IBA and Clonex, respectively. However, these rooting percentages were not significantly different from the 67% obtained without a rooting inductor. However, we can see significant differences in the number of roots, with a great advantage with the Clonex treatment, which can have an impact on acclimatization performance. In *P. tridentatum*, the best rooting percentage was also obtained with Clonex (90%), with a significant difference from the control (73%). Also, significant differences in the number of roots were recorded between the treatments and the control, with an advantage for the former. The choice of ex vitro rooting is almost always advantageous, as it generally reduces costs and allows for more functional root systems. 

Moreover, the presence of a growth regulator used in the MM medium influences rooting. Since rooting is spontaneous, no auxin addition is necessary. By observing our ex vitro rooting results, it is possible to state that *L. stoechas* subsp. *luisieri* could be rooted in the absence of growth regulators. In the Lamiaceae family, the in vitro rooting of two *Salvia* spp. in the absence of growth regulators resulted in a high rooting percentage compared to IBA or IAA [26]. 

On the other hand, dipping in 100 mg L^−1^ NAA for 1 min was found to be the best treatment for the ex vitro rooting of *Siratia grosvenorii* when compared to IBA (100 mg L^−1^) [27]. In the same study, the authors showed that ex vitro rooting was superior to the in vitro process, with a well-developed root system and higher rooting and transplant survival rates [27]. Furthermore, in *Salvia officinalis*, the best in vitro rooting was achieved with 0.9 mg L^−1^ IAA [28]. In the previous study, the authors also investigated the ex vitro rooting of *S. officinalis* and reported that IBA promotes higher rooting rates in the spring. In addition to some studies on Lamiaceae family species that found the best rooting rates without growth regulators, it has been reported that using auxins promotes their formation [27,28,29]. 

Having achieved a robust protocol for the micropropagation of these two species, we consider it of the utmost importance to associate in vitro conservation methodologies that ensure their preservation. Using in vitro methods has been progressively gaining ground over other conventional preservation methods since it has been possible to develop highly efficient protocols. Two basic approaches are followed to maintain germplasm collections in vitro: (i) minimal growth and (ii) cryopreservation [30]. Minimal growth conditions for short- to medium-term storage can be established in several ways: reduced temperature and light; the incorporation of sub-lethal levels of growth retardants; the induction of osmotic stress with sucrose or mannitol; the maintenance of cultures at a reduced nutritional status; notably reduced carbon; the reduction of gas pressure over the cultures; and desiccation and mineral oil overlay. The advantage of this approach is that cultures can be readily brought back to normal culture conditions to produce plants on demand. Generally, for temperate species, the storage temperature ranges from 2 to 5 °C [31]. The low temperatures are also combined with a decrease in light intensity or total darkness. The medium composition is another factor that could be used for long conservation with full or reduced strength of its salts. The osmotic gradient, such as the sucrose concentration, can also be reduced; the most common is 2% instead of the standard 3% [32].

In our experiments, the viability of *L. stoechas* subsp *luisieri* was achieved after 12 months of cold conservation at both sucrose concentrations (15 and 30 g L^−1^), with survival rates higher than 80%. Also, in *P. tridentatum*, the explant’s viability was higher than 90% for the same period of slow-growth conservation. In a Lamiaceae species, *Thymbra spicata*, in vitro conservation with sucrose reduction showed better survival results than high sucrose concentrations during 12 weeks of conservation [33]. Under standard light conditions for 6 months at 18 °C, *Ceratonia siliqua* (Fabaceae family) explants showed a survival rate of 100% [34]. In Rosaceae family species, the use of reduced temperatures of 4 °C, with sucrose reduction to 2%, in darkness for 12 months, led to a 99% survival rate on *Crataegus monogyna* and *Cydonia oblonga* explants [32]. Also, the composition of media contributes to the success of the survival rate, namely the reduction of the strength of salts and the sucrose concentration. Another critical factor is the use of growth regulators, which has been discussed in the literature [7].

## 4. Materials and Methods

### 4.1. Plant Material 

In vitro shoot cultures of *L. stoechas* subsp. *luisieri* were established from single-node explants of the selected greenhouse plants. These plants were obtained by cuttings from one adult field plant in Serra da Malcata, Penamacor, Portugal (coordinates of 40°12′06.741 N, 7°06′22.085 W). *P. tridentatum* also has its origin in Serra da Malcata (coordinates pf 40°14′05.942 N, 7°06′52.804 W). This species has already been established in vitro previously, as described by Gonçalves at al. [35]. The voucher specimens were deposited at the herbarium of the Biology Laboratory of IPCB-ESA (Polytechnic Institute of Castelo Branco-Agrarian School), with voucher numbers ESACBMLS08 and ESACBPTM01 for *L. stoechas* subsp. *luisieri* and *P. tridentatum*, respectively. Plants were collected following good agricultural and collection practices to ensure natural regeneration and propagation of the species. The plants were collected so that the area’s biodiversity and ecological balance were not compromised. Only a portion of the plant was collected, and to avoid contamination, collection, the collection took place only from healthy, disease-free plants and not in over-exploited areas [36].

### 4.2. In Vitro Establishment and Multiplication

Young branches of *L. stoechas* subsp. *luisieri* were washed first under running tap water for 30 min, followed by a 2 g L^−1^ fungicide solution (Benlate, Du Pont Iberica, Barcelona, Spain) for 10 min. After, shoots were immersed in 70% (*v*/*v*) ethanol for 30 s and then surface-sterilized with 1% and 2% (*v*/*v*) sodium hypochlorite (NaOCl) solutions containing a few drops of polyoxyethylenesorbitan monolaurate (Tween-20) for 10 and 15 min (E1: 1% NaOCl for 10 min; E2: 1% NaOCl for 15 min; E3: 2% NaOCl for 10 min; E4: 2% NaOCl for 15 min), following rinsing in sterile water (three washes, 3 min each). The node segments were cut to appropriate sizes (1–1.5 cm) and placed in test tubes (25 mm diameter × 150 mm height) with MS medium [19] supplemented with 3% sucrose, 0.7% agar, and 0.2 mg L^−1^ BAP and pH-adjusted to 5.7–5.8 with 1 N HCl or 1 N NaOH. The medium used for the *L. stoechas* subsp. *luisieri* shoot multiplication was the same as previously mentioned (MM, multiplication medium). The conditions chosen were the results of previous experiments (unpublished). For *P. tridentatum,* the plant material used were shoots already being multiplied in vitro, and the culture medium for shoot multiplication was the MS medium supplemented with 1 mg L^−1^ BAP, 0.5 mg L^−1^ IBA, 3% sucrose, and 0.7% agar, and the pH was adjusted to 5.5–5.6 (MM, multiplication medium). These culture conditions were chosen based on previous work [37]. The explants from both species were cultured in glass flasks (65 mm diameter × 85 mm high) with 50 mL of medium and 7 explants each. All cultures were incubated in a growth chamber under a 16/8 h photoperiod at 25/22 °C, respectively, with neutral-white fluorescent lamps (OSRAM, L58W/840) at a 50 μmol m^−2^ s^−1^ photosynthetic photon flux density (PPFD). Shoots were subcultured at 4–5 weeks intervals in the same physical conditions described for establishment. The number of new shoots, length of the longest shoot (cm), multiplication rate, fresh weight (mg), dry weight (mg), hyperhydricity (%), and mortality (%) were registered after 50 days in culture. The multiplication rate was calculated by determining the number of new secondary explants produced from a single explant.

### 4.3. Ex Vitro Rooting

Before ex vitro rooting treatments, the shoots with a length of ≥2 cm were transferred to an MS hormone-free culture medium for 20 days. For root induction, three treatments were performed: without hormone (control), by dipping the basal part of the shoots for 45 s in 1 g L^−1^ of IBA, or in Clonex^®^ (3.3 g L^−1^, Growth Technology, Taunton, UK). These two rooting conditions were chosen on the basis of previous experiments (unpublished). The shoots were placed in a sterilized brown peat: perlite (1:2) substrate in 60 × 40 × 20 cm polystyrene boxes. The boxes were covered with transparent acrylic plastic. The shoots were sprayed weekly with a nutritive solution (Murashige and Skoog salt solution with macronutrients at half strength and nitrates at quarter strength, pH at 5.7) and placed in a growth chamber under the same conditions described for the multiplication phase, except the PPFD at 150 µmol m^−2^ s^−1^. After eight weeks, the rooting rate (%), root number per shoot, and length of the longest root (cm) were recorded.

### 4.4. Slow-Growth Conservation Assay

For prolonged storage, 1.5–2 cm long explants were isolated from in vitro shoots and cultured in the MM medium previously described for each species. Three sucrose concentrations, 0, 15, and 30 g L^−1^, were tested. The cultures were stored in the dark at 4 °C for 12 months, with the morphological parameters measured every three months. Preceding their evaluation, the explants were maintained in a growth chamber for two days before being subcultured in a new MM medium containing the standard sucrose concentration (30 g L^−1^). After four weeks, the survival rate, number of new explants, length of the longest shoot (cm), and multiplication rate were assessed. These parameters were assessed for three successive subcultures. 

### 4.5. Statistical Analysis

For the establishment experiments, two replicates of 30 explants were used per treatment. For the multiplication experiments, 30 explants were used per treatment, and the experiment was evaluated in 2 successive subcultures. For the slow-growth conservation experiments, 30 explants were used per treatment, and the experiment was evaluated in 3 consecutive subcultures. The program SPSS for Windows v.25 was used to perform an analysis of variance (ANOVA), and the means (±se) were compared at the *p* ≤ 0.05 level of significance using Duncan’s multiple range test. The percentage data were transformed into arcsin values prior to analysis. 

## 5. Conclusions

This paper describes a protocol for the micropropagation and in vitro slow-growth conservation of *L. stoechas* subsp. *luisieri* and *P. tridentatum*. The high multiplication rates and successful ex vitro rooting contribute to the ability to propagate these species for a range of applications, including research, conservation, and commercial uses. The slow-growth conservation protocol provides a way to preserve genetic material over long periods, reducing labor, energy, and reagent costs, which is crucial for in vitro biodiversity conservation. 

## Figures and Tables

**Table 1:** Number of new shoots (Nns), length of the longest shoot (Ls, cm), and multiplication rate (Mr) of both species in the multiplication phase.

	Nns	Ls (cm)	Mr
*L. stoechas* subsp. *luisieri*	3.3 ± 0.3	4.9 ± 0.2	6.8 ± 0.3
*P. tridentatum*	6.8 ± 0.4	5.1 ± 0.1	13.3 ± 0.8

The results are expressed as the means ± standard errors.

**Table 2:** Effects of different rooting treatments in *Lavandula stoechas* subsp. *luisieri* and *Pterospartum tridentatum* explants.

*L. stoechas* subsp. *luisieri*				*P. tridentatum*		
	Rooting Rate (%)	Number of Roots	Length of the Longest Root (cm)	Rooting Rate (%)	Number of Roots	Length of the Longest Root (cm)
IBA	73 ^a^	8.0 ± 5.1 ^b^	6.8 ± 2.6 ^b^	83 ^ab^	2.1 ± 1.0 ^a^	3.2 ± 1.3 ^a^
Clonex^®^	77 ^a^	15.0 ± 6.5 ^a^	8.1 ± 1.6 ^a^	90 ^a^	2.1 ± 0.9 ^a^	3.6 ± 1.2 ^a^
Control	67 ^a^	5.0 ± 2 ^b^	6.4 ± 2.6 ^b^	73 ^b^	0.9 ± 0.6 ^b^	3.6 ± 1.3 ^a^

The results are expressed as the means ± standard deviations. Different letters in the same column indicate significant differences (*p* < 0.05).

## Data Availability

The data used in this study are reported in the paper’s figures and tables.
